# Documentation of atrial fibrillation among non-conveyed ambulance patients: a new primary prevention opportunity?

**DOI:** 10.29045/14784726.2022.06.7.1.51

**Published:** 2022-06-01

**Authors:** Emily Heppenstall, Graham McClelland, Chris Price, Chris Wilkinson

**Affiliations:** Newcastle University ORCID iD: https://orcid.org/0000-0003-2423-6221; Newcastle University; North East Ambulance Service NHS Foundation Trust ORCID iD: https://orcid.org/0000-0002-4502-5821; Newcastle University ORCID iD: https://orcid.org/0000-0003-3566-3157; Newcastle University ORCID iD: https://orcid.org/0000-0003-0748-0150

**Keywords:** ambulance, atrial fibrillation, stroke

## Abstract

**Introduction:**

Atrial fibrillation (AF) is the most common sustained cardiac arrhythmia and is a significant risk factor for stroke. Prescription of oral anticoagulant (OAC) medication reduces the risk of AF-related stroke by 64% – yet over 400,000 people in England have undiagnosed (and therefore untreated) AF.

Emergency medical services (EMS) encounter a wide range of patients, some of whom may not engage with other healthcare services. AF may be detected by EMS in connection with the cause of the call, or as an incidental finding. While EMS are not traditionally utilised for public health screening, they may offer an opportunity to identify patients with undiagnosed or untreated AF and refer onward.

This study aimed to explore what proportion of patients seen by EMS who were not transported to hospital had AF and to estimate how many would potentially benefit from OAC.

**Methods:**

A retrospective service evaluation was conducted using routinely collected data from a large UK regional ambulance service. The sample included adults attended by EMS on the 15th of each month in 2019, who were not transported to hospital and where an electrocardiogram was recorded. Of those with AF, we calculated the proportion in whom this was possibly new and report whether OAC was prescribed.

**Results:**

There were 859 patients who met the inclusion criteria, of whom 91 (11%) had AF documented. Of the 91 patients with AF, 23 (25%) had no documented history of AF or OAC prescription, so were potentially new diagnoses of AF, who would benefit from consideration of OAC therapy.

**Conclusion:**

The EMS assessment offers an opportunity for AF to be identified in patients who were not transported to hospital. EMS may have a role in primary prevention of harm, including stroke, by identifying and referring patients with AF for consideration of OAC.

## Introduction

Atrial fibrillation (AF) is the most common sustained cardiac arrhythmia and is associated with an increased risk of stroke and systemic embolism (Lippi et al., 2020). The risk of stroke is substantially reduced by the prescription of oral anticoagulant (OAC) medications in people with AF, yet approximately one third of patients with AF in England have not yet had their arrhythmia recognised, and so have not had the opportunity for consideration of OAC – and therefore remain at elevated risk of stroke (Public Health England, 2020). It is necessary to maximise opportunities for AF detection in order to facilitate OAC prescription and reduce the number of avoidable strokes.

Emergency medical services (EMS) attend a population representing all demographic groups, collect rich clinical data on the patients they encounter and potentially encounter patients who do not regularly engage with healthcare either due to a lack of need for healthcare or other barriers (Phung et al., 2015). Most patients attended by EMS are conveyed to hospital for further medical assessment, investigations and treatment. However, in the United Kingdom a growing number of calls to EMS do not result in the patient being conveyed to a hospital. Indeed, 32% of patients attended in June 2021 by EMS in England were not transferred to hospital (NHS England, 2021). Instead, patients were treated at the scene by EMS staff, perhaps with access to alternative care pathways through primary care. However, we lack data on the care processes and clinical outcomes for this ‘non-conveyed’ population (O’Cathain et al., 2018).

This service evaluation explored what proportion of the patients attended by the North East Ambulance Service NHS Foundation Trust (NEAS) who were not transported to hospital were in AF at the time of their assessment, whether this diagnosis appeared to be new, whether they were prescribed OAC in the context of their future risk of cardioembolic events, as well as important characteristics of the emergency calls (initial complaint and reason for non-conveyance). Our aims were to explore the proportion of patients with AF who were not prescribed OAC who might benefit, and whether EMS provide a novel health screening opportunity to detect AF.

## Methods

This observational retrospective service evaluation used routinely collected data extracted from electronic patient care records (EPCRs).

### Setting

NEAS is one of 10 regional ambulance services in England. NEAS serves Northumberland, Tyne and Wear, County Durham, Darlington and Teesside, comprising a population of over 2.7 million, and manages over 1.5 million calls per year. Ambulance crews include two staff members: usually one paramedic and one non-paramedic.

### Data and participants

Data were extracted for calls to NEAS between 00:00 and 23:59 on the 15th day of every month in 2019. This sampling strategy was designed to overcome any seasonality effect, and the impact of COVID-19. Patients were included if they were 18 years of age or older, not transported to hospital and if an electrocardiogram (ECG) was documented. Patients in cardiac arrest were excluded from the evaluation, but all other dispatches were included.

The following data were extracted manually from the NEAS EPCR for each participant: sex; reason for call; reason for non-conveyance; crew composition; initial observations (heart rate and systolic blood pressure); ECG report and recording; components of the CHA_2_DS_2_-VASc stroke risk score (congestive heart failure, hypertension, age, diabetes, stroke / transient ischaemic attack, vascular disease) (Lip et al., 2010); and medication relevant to treatment of cardiovascular diseases including hypertension, hypercholesterolaemia, heart failure, stroke and ischaemic heart disease.

### Identification of AF

Identification of AF was taken from the NEAS crew documentation in the EPCR or the automated reports generated by a standard ECG device (Zoll X-series defibrillators). ECGs uploaded to the EPCR were screened by EH to validate the automated reports, with unclear ECGs flagged for adjudication by CW (NIHR academic clinical lecturer in cardiology) and GM (research paramedic). AF was diagnosed in accordance with international guidelines for diagnosis of AF, which require ‘a standard 12-lead ECG recording showing heart rhythm with no discernible repeating p waves and irregular RR intervals’ (European Society of Cardiology, 2020). If there was no documentation of AF by the NEAS crew and no AF identified on the ECG, then the patient was classified as not having AF. Patients were classified as potentially new AF if there was no medical history of AF and no OAC prescription recorded at any point in the ambulance documentation, including the free-text record of clinical assessment in the EPCR.

The CHA_2_DS_2_-VASc score was used to estimate the annual risk of stroke in patients with AF. Scores range from zero to nine (higher scores indicating greater stroke risk). We used thresholds for treatment based upon guidance from the National Institute for Health and Care Excellence. These suggest that OAC prescription should be considered in men with a CHA_2_DS_2_-VASc score of one and prescribed in men or women with a score of two or more, alongside an assessment of bleeding risk (National Institute for Health and Care Excellence, 2021).

### Statistical methods

Data were analysed using SPSS Statistics Version 27, Microsoft Excel and Graph Pad Prism 9. Descriptive statistics were reported, with median and interquartile range for non-normally distributed data and mean and standard deviation for normally distributed data. For comparing populations, Mann Whitney U tests were used for non-normal continuous numerical data. Chi square tests were used for categorical variables. Fisher’s exact tests were used for smaller samples where the expected cell counts did not exceed five. The prevalence of AF in the non-conveyed, adult population with 95% confidence interval (95% CI) was calculated. The statistical significance threshold was set at p < 0.05. All data were collected on a positive recording basis (whereby the absence of a recorded diagnosis is treated as the absence of that event). Therefore, no formal missing data strategy was employed.

### Patient and public involvement

There was no patient or public involvement in this study.

## Results

### Identification of AF

In total, 2433 calls resulted in non-conveyance over the 12 days. Of these, 859 (35.3%) had an ECG recorded. An ECG displaying AF was present in 67 patients, and in 24 patients the presence of AF was documented on the EPCR but the ECG was not attached. We found no statistically significant difference in the sex, age or initial observations between those with AF and an ECG available and those with AF and no ECG. In total, 91 patients had AF documented, therefore the prevalence of AF in the non-conveyed, adult population was 10.5% (95% CI 8.5–12.7).

The participant characteristics are reported in Table 1 by AF status (AF or no AF). There were minimal (< 1%) missing data in demographics and 5% of patients had no past medical history documented. Medication data were absent for 16% of patients.

**Table 1.** Baseline characteristics of participants.

Variable	All patients, n = 859 n (%)	Patients with AF, n = 91 n (%)	Non-AF patients, n = 768 n (%)	Difference between AF and non-AF patients
**Demographics**				
Male sex	399 (46.4)	56 (61.5)	343 (44.7)	< ***0.01*†**
Age, median (IQR)	67 (42–82)	83 (75–87)	63 (38–81)	< ***0.01*†**
Heart rate (bpm), median (IQR)	83 (73–95)	84 (74–93)	83 (73–95)	0.58†
Systolic BP (mmHg), median (IQR)	135 (120–153)	137 (115–155)	135 (120–153)	0.86†
**Past medical history of cardiovascular disease**				
Diabetes	143 (16.6)	24 (26.4)	119 (15.5)	***0.01*^**
Heart failure	46 (5.4)	17 (18.7)	29 (3.8)	< ***0.01*^**
Hypertension	208 (24.2)	37 (40.7)	171 (22.3)	< ***0.01*^**
Stroke/TIA	69 (8.0)	11 (12.1)	58 (7.6)	0.16^
Vascular disease	142 (16.5)	22 (24.2)	120 (15.6)	***0.04*^**
**Cardiovascular medication**				
Angiotensin-converting enzyme inhibitors / angiotensin-receptor blockers	61 (7.1)	12 (13.2)	49 (6.4)	< ***0.01*^**
Anti-anginal	45 (5.2)	9 (9.9)	36 (4.7)	***0.02*^**
Anti-platelets	104 (12.1)	12 (13.2)	92 (12.0)	0.67^
Beta blockers	106 (12.3)	27 (29.7)	79 (10.3)	< ***0.01*^**
Calcium-channel blockers	54 (6.3)	11 (12.1)	43 (5.6)	***0.01*^**
Cholesterol-lowering medication	180 (21.0)	37 (40.7)	143 (18.6)	< ***0.01*^**
Diuretics	35 (4.1)	10 (11.0)	25 (3.3)	< ***0.01*^**
OAC	83 (9.7)	49 (53.8)	34 (4.4)	< ***0.01*^**

†Mann Whitney U test; ^Chi square test.

AF: atrial fibrillation; BP: blood pressure; IQR: interquartile range; OAC: oral anticoagulation; TIA: transient ischaemic attack.

### Potential new AF

Of the 91 patients with AF, 62 (68.1%) patients had a documented history of AF and 49 (53.8%) patients had OAC prescription documented. From the 29 patients with no documented history of AF, six (20.7%) were already prescribed OAC and therefore excluded as possible new AF, suggesting that the diagnosis of AF was potentially new for 23 individuals.

### Estimated stroke risk of participants with AF

The CHA_2_DS_2_-VASc score was calculated for the 91 patients with AF. Of the 35 women, 34 (97.1%) had a CHA_2_DS_2_-VASc score of two or more (and were therefore considered eligible for OAC prescription). Of these, 20 (58.8%) were documented to be prescribed OAC. Of the 56 men with AF, all were at or above the recommended threshold for considering OAC prescription (CHA_2_DS_2_-VASc of one or more), and OAC prescription was documented in 29 (51.8%). In total, 41 (45.1%) AF patients, including all 23 potentially newly diagnosed patients, with a CHA_2_DS_2_-VASc score at or above their treatment threshold were not prescribed OAC.

### Initial complaint

There was a wide range of presenting complaints reported at the initial contact with EMS among the 859 study participants. Overall, the most common complaints were of chest pain (n = 173, 20.1%) and shortness of breath (n = 129, 15.0%). Among patients with AF, the most common complaints were: shortness of breath (n = 16, 16.7%); chest pain (n = 12, 13.2%); and falls (n = 12, 13.2%). In those without AF, these were: chest pain (n = 161, 21.0%); falls (n = 113, 14.7%); and shortness of breath (n = 71, 9.4%).

### Reasons for non-conveyance

The most frequent reason for non-conveyance to hospital was an EMS decision that adequate assessment and treatment had been provided on-scene (n = 331, 38.5%, Table 2). In the 158 (18.4%) cases where the patient was referred to another service, the most frequent services used were: GP (n = 124, 78.4%); urgent care centre (n = 10, 6.3%); and the falls service (n = 6, 3.8%).

**Table 2.** Reasons for non-conveyance.

Reason for non-conveyance	Non-AF patients, n = 768 n (%)	Patients with AF, n = 91 n (%)
Crew decision: treated on-scene	293 (38.9)	38 (41.8)
Advice to contact GP	156 (20.7)	16 (17.6)
Referred to another service	138 (18.3)	20 (22.0)
Refusal to travel	128 (17.0)	11 (12.1)
Emergency healthcare plan in place	15 (2.0)	4 (4.4)
Patient making their own way	23 (3.1)	2 (2.2)
Not recorded	15 (1.9)	0 (0)

## Discussion

Our work shows that there is the potential for EMS clinicians to play a role in identifying patients with AF that may benefit from further evaluation and consideration of treatment with OAC to reduce their risk of stroke. In patients who were assessed but not transported to hospital by ambulance in North East England, we found that approximately 1 in 10 patients had AF. The AF appears to be a new diagnosis in 23 patients over 12 days, suggesting that NEAS could potentially identify up to two patients per day with undiagnosed and therefore untreated AF.

Although no previous studies have examined the burden of AF in the non-conveyed population or their clinical characteristics, we report similar age and sex demographics to other non-conveyed populations (Ebben et al., 2019; Forsgärde et al., 2020; Höglund et al., 2020; Lederman et al., 2020; Vloet et al., 2018). The study population was defined by the recording of an ECG, which may have contributed to a higher median age than an unselected population (Lederman et al., 2020). We found that in comparison to the non-AF population, those with AF were older, more commonly men and had a greater prevalence of cardiovascular comorbidities including hypertension, heart failure, diabetes and vascular disease. This is concordant with the known epidemiology of AF (Kirchhof, 2017).

We found that the burden of AF in the non-conveyed study population was 10.6%. While this is higher than the general UK prevalence of up to 3.3% (Adderley et al., 2019; Zoni-Berisso et al., 2014), the median age of our study population is 67 years. Our estimate is concordant with a primary care population with a similar age profile: the prevalence of known AF in people aged 65 years or older is reported at 11.4% (Wilkinson et al., 2021).

This study identified breathlessness and chest pain as the most common initial presenting complaints. While other studies have found that neurological or trauma-related conditions were the most frequent initial complaints in a general population (Ebben et al., 2017), this difference may be explained by the inclusion requirement of an ECG in this study.

We found that a lower proportion of non-transported patients were prescribed OAC than one previous report (9.7% vs. 28%) (Simpson et al., 2014). However, this may be explained by differences in the inclusion criteria between the two studies, as Simpson et al. (2014) focused on non-conveyed patients with falls.

### Strengths and limitations

This study addresses an important knowledge gap by reporting the burden of AF among other cardiovascular risk factors in the non-conveyed EMS population. Our sampling strategy accounted for potential daily, weekly and seasonal variation which may impact on the rates of non-conveyance (Hoikka et al., 2017; Vloet et al., 2018), and excluded the COVID-19 era. Every available ECG was reviewed with a robust adjudication process against standard criteria for the diagnosis of AF, so the burden of AF is unlikely to have been overestimated; however, the presence of an irregular pulse may have led to the clinician being more likely to record an ECG which would affect the population included. A robust, widely used and clinically validated tool was used to calculate the estimated annual stroke risk.

However, we recognise the limitations of our work. The study is reliant on clinical documentation for the past medical and medication histories, which may have been incomplete or incorrect for some patients. There is therefore the possibility of misclassification of patients’ AF as new, when in fact it was known to the GP (but not the EMS crew). As primary and secondary care records are becoming increasingly accessible to paramedics, missing data should become less of an issue in future. The burden of AF in this study may have been underestimated, as approximately one third of the non-conveyed population had ECGs attached to their health record, and patients with paroxysmal AF may have been in sinus rhythm at the time of the recording. Furthermore, the ECG recording was a 12-lead which did not fulfil the full definition of > 30 seconds, but this did reflect current paramedic practice. Finally, we recognise that while this is the largest study of its kind to date, the sample size remains relatively small and still vulnerable to external effects upon case selection.

### Implications for research

In this study, we did not have access to data to indicate whether OAC had already been considered in the population with AF. OAC may have already been considered and deemed to be contra-indicated, inappropriate or declined by the patient in these cases. Further research is therefore needed using a dataset linked to primary and secondary care records in order to establish whether the diagnosis of AF is already known, and whether OAC has previously been considered. Secondly, work is needed to investigate the effectiveness of current referral pathways for non-transported patients with incidental findings such as AF and how often such referrals lead to changes in patient care. Finally, we need to understand more about the acceptability and possible unintended consequences of screening for additional medical conditions in an EMS setting. This would require a more qualitative approach, engaging with patients, primary care providers and other stakeholders.

### Implications for public health

Prevention of stroke was identified as the number one priority in a recent research priority-setting exercise by the James Lind Alliance (Stroke Priority Setting Partnership, 2021). Preventing strokes through OAC treatment of people with AF would lead not only to improved outcomes for patients but also to potentially significant financial savings for the NHS. On average, the societal cost of stroke is £45,409 per person in the first 12 months after stroke – and £26 billion overall annually (Patel et al., 2018, 2020). This suggests that interventions that reduce the incidence of stroke are likely to yield substantial economic savings to society, as well as reducing avoidable morbidity and mortality.

People who access emergency care may differ in their care-seeking behaviour and have fewer interactions with primary care than people accessing other healthcare services, so EMS clinicians may be uniquely placed to identify AF and facilitate a holistic assessment of the risks and benefits of OAC through an appropriate referral pathway. There may also be scope for an extended role for EMS in facilitating primary prevention with regard to a greater range of health conditions, making use of their access to a sometimes ‘hard to reach’ population to make every contact count (Health Education England, 2021). While this study was completed using data from one ambulance service, the concept is generalisable to other ambulance services which have the capability to identify AF and refer patients for incidental findings such as AF.

## Conclusions

This prevalence of AF in a non-conveyed population in which an ECG was recorded was 10.6%. This was a possible new diagnosis for two patients per day who are likely to benefit from assessment for consideration of OAC to reduce their future risk of stroke. EMS may therefore have an important role in stroke prevention through identification of modifiable risk factors including AF, which will reduce the morbidity, mortality and economic burden caused by stroke.

## Author contributions

EH and GM are joint first authors. EH collected and analysed the data and wrote the original report; GM, CP and CW developed the study and supervised EH throughout; all authors contributed to the manuscript and approved the final version. EH acts as the guarantor for this article.

## Conflict of interest

GM is the editor-in-chief of the *BPJ*.

## Ethics

This project was a service evaluation, so no formal ethical approval was needed. The project was approved by Newcastle University (23 March 2021) and registered as a service evaluation with NEAS (ref. NEAS-SE-04-2021, 1 April 2021). Caldicott approval was secured from NEAS for data sharing.

## Funding

None.
